# Ventriculoperitoneal Shunt Peritoneal Catheter Knot Formation

**DOI:** 10.1155/2013/628493

**Published:** 2013-09-15

**Authors:** Anwar Ul-Haq, Faisal Al-Otaibi, Saud Alshanafey, Mohamed Diya Sabbagh, Essam Al Shail

**Affiliations:** ^1^Division of Neurosurgery, Neurosciences Department, King Faisal Specialist Hospital and Research Center, P.O. Box 3354, Riyadh 11211, Saudi Arabia; ^2^College of Medicine, Alfaisal University, King Faisal Specialist Hospital and Research Center, Riyadh 11533, Saudi Arabia; ^3^Division of Pediatric Surgery, Department of Surgery, King Faisal Specialist Hospital and Research Center, Riyadh 11211, Saudi Arabia

## Abstract

The ventriculoperitoneal (VP) shunt is a common procedure in pediatric neurosurgery that carries a risk of complications at cranial and abdominal sites. We report on the case of a child with shunt infection and malfunction. The peritoneal catheter was tethered within the abdominal cavity, precluding its removal. Subsequently, laparoscopic exploration identified a knot at the distal end of the peritoneal catheter around the omentum. A new VP shunt was inserted after the infection was healed. This type of complication occurs rarely, so there are a limited number of case reports in the literature. This report is complemented by a literature review.

## 1. Introduction

Ventriculoperitoneal (VP) shunt insertion is one of the most common procedures performed in neurosurgical practice. The abdominal complications of VP shunt insertion include cerebrospinal fluid (CSF) ascites, loculated cysts, hydrocele, infection, shunt extrusion, shunt migration, CSF leaks, viscous perforations, and protrusion of the catheter from the anus [1, 2]. Spontaneous knotting of the peritoneal catheter is a rare complication of the VP shunt [3]. Here, we report a case of knotting of the peritoneal catheter discovered during the removal of a malfunctioning VP shunt. The knotting of the catheter hindered its removal; the catheter was later removed laparoscopically.

## 2. Case Report

**Figure 1:**
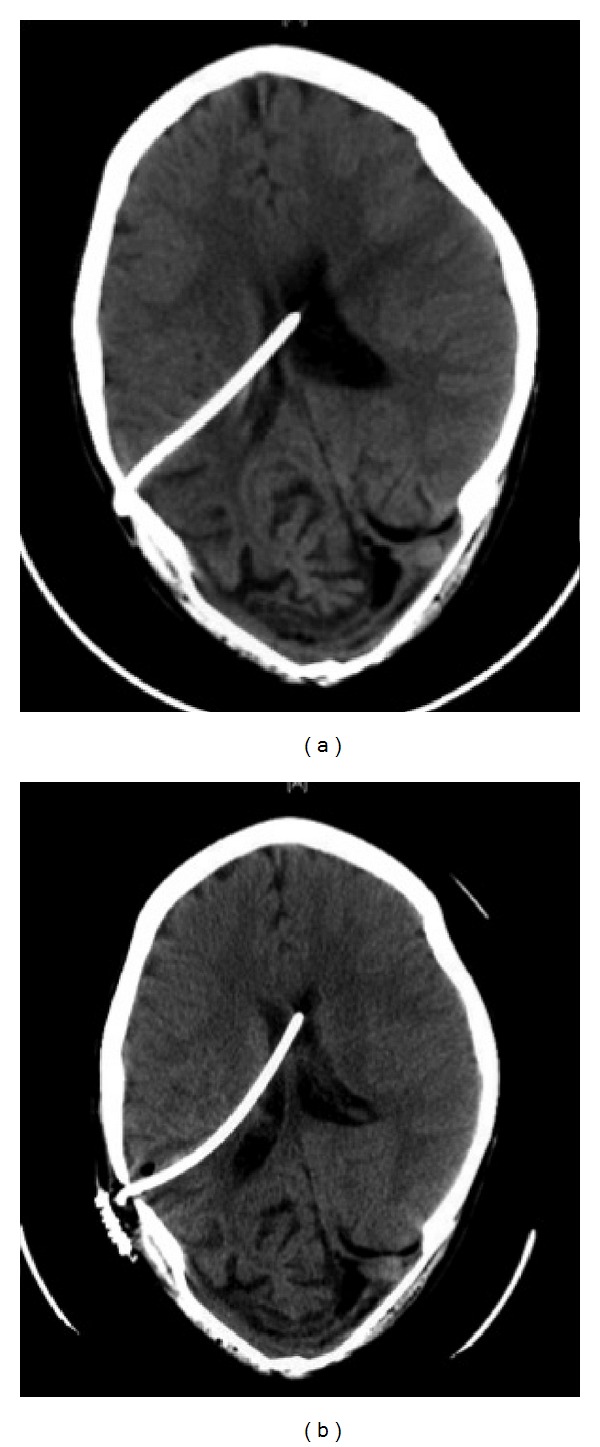
Computed Tomography of the brain demonstrating dilated ventricular system as a sign of ventriculoperitoneal shunt malfunction (a) and the reduction in ventricular size after VP shunt revision (b).

**Figure 2:**
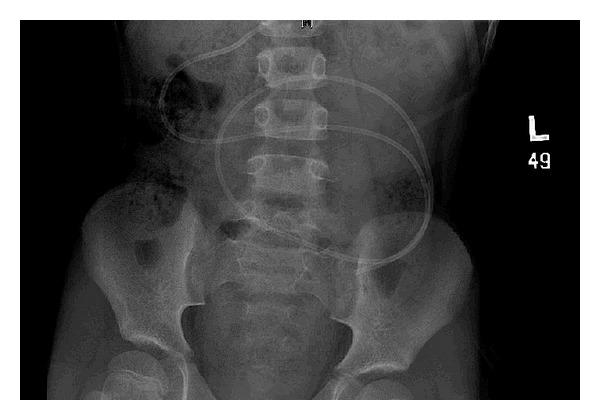
Abdominal X-ray showing the peritoneal catheter with early large knot formation.

**Figure 3:**
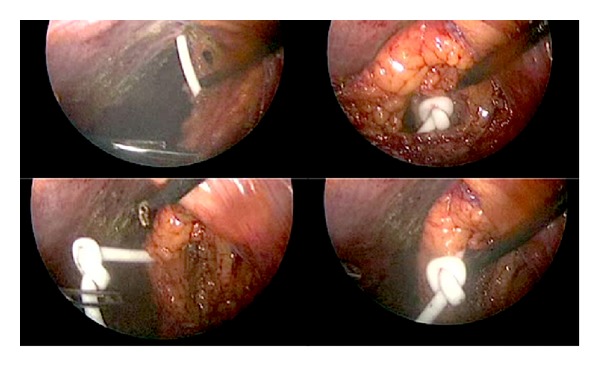
Laparoscopic view depicting the knot formation at the distal peritoneal catheter embedded within omentum.

This particular case refers to an eight-year-old boy who was born with a congenital hydrocephalus and large parieto-occipital skull defect. He underwent VP shunt insertion after birth. He then underwent cranioplasty with titanium mesh and acrylic bone cement on January 20, 2009. One month later, he was presented to the emergency room with a severe headache and blurred vision. His ventricular catheter had migrated out of the ventricle, and the shunt was not functioning. An emergency external ventricular drain was inserted, which was replaced later with a VP shunt. A peritoneal catheter was inserted laparoscopically. Two years later, the boy returned with a headache, vomiting, fever, and seizures. The results of a CSF analysis indicated infection, and a Computed Tomography (CT) scan of his brain showed hydrocephalus, suggesting a shunt malfunction (Figure 1). A VP shunt X-ray series showed that the peritoneal catheter was coiled in the abdomen (Figure 2). He underwent removal of the VP shunt and insertion of an external ventricular drain. The ventricular catheter was removed easily prior to the insertion of the external drain. During removal, it was noted that the peritoneal catheter was difficult to remove and felt tethered at the abdominal entrance site. The upper part of the peritoneal catheter was cut and removed, and the remaining part was left in place to be dealt with later. Once the CSF infection cleared, the patient again underwent VP shunt insertion with the laparoscopic-assisted approach. During the procedure, it was noticed that there was a knot at the distal end of the peritoneal catheter, and the catheter was stuck at the inner surface of the abdominal wall near the point of its entrance (Figure 3). The omentum adhered to the abdominal wall at the point of entrance of the peritoneal catheter. The peritoneal catheter was removed through a separate port, and a new VP shunt was implanted. Subsequently, the patient did well and was discharged home in a healthy state.

## 3. Discussion

VP shunt implantation is the most common surgical procedure used to treat hydrocephalus. This procedure has a variety of complications, including shunt obstruction, infection, fracture, disconnection, migration, subcutaneous extrusion, or protrusion of the catheter. These problems may relate to the ventricular catheter, shunt reservoir, or peritoneal catheter. Abdominal complications are reported in 5–47% of VP shunt cases [1]. Such complications include CSF ascites, hydrocele, loculated peritoneal cysts, bowel perforations, peritoneal catheter obstruction by omental adhesions, subcutaneous extrusion of the peritoneal catheter, CSF fistula, incisional hernia, and protrusion of the peritoneal catheter from the anus [1, 4–8]. Knot formation on the peritoneal catheter is an extremely rare complication of VP shunt insertion [9, 10]. To date, there are a limited number of case reports available.  Table 1 summarizes the reported cases of peritoneal catheter knot formation.

The formation of a knot in a peritoneal catheter commonly results in VP shunt malfunction [3, 9, 11–13]. Knotting of the catheter around the bowel can lead to bowel obstruction and gangrene [4, 14]. Occasionally, the knot is an incidental finding [10]. The issue can be diagnosed by a VP shunt X-ray series and CT scan. Treatment consists of laparoscopic exploration of the peritoneal cavity or minilaparotomy. The peritoneal catheter can be unknotted or removed and replaced with a new catheter; alternatively, the whole shunt system can be replaced [15].

Knot formation usually occurs at the terminal end of the peritoneal catheter. The exact mechanism of knot formation is not clearly known. The various factors proposed by different authors include catheter characteristics, capacity and configuration of abdominal cavity, and direction of catheter movement [11]. A catheter's greater length, lesser diameter, and highly elastic material predispose it to knotting. Increased abdominal volume, crowding of abdominal contents, intra-abdominal adhesion, and vigorous peristalsis can also trigger knot formation [10]. Raymer and Smith reported on the mechanism of knot formation [16]. The researchers placed a string perpendicular to the pull of gravity within a rotating cubic box, causing the string to form a knot. The authors found that the length of string and the increase in string motion raise the probability of knot formation. In our case, knot formation occurred during the removal of the peritoneal catheter due to the adhesions of the omentum to the point of entry of the peritoneal catheter. 

## 4. Conclusion

Knotting of the peritoneal catheter is a rare complication of the VP shunt. The exact mechanism of knot formation remains poorly understood. Asymptomatic knots on the peritoneal catheter can be observed with serial VP shunt X-ray series and a CT scan, and symptomatic patients require laparoscopic exploration and the unknotting or total replacement of the peritoneal catheter.

## Figures and Tables

**Table 1:** Summary of reported cases of peritoneal catheter knot formation.

Author/year	Age/sex	Basic pathology	Time interval between shunt and presentation	Presentation	Management
Starreveld et al. (1998) [4]	7 days/F	Hydrocephalus	NA	Bowel gangrene	Bowel resection and ventriculoatrial shunt
Toshifumi and Tatsuro (2001) [14]	63 Y/M	Head injury and hydrocephalus	20 years	Bowel obstruction without gangrene	Laparotomy and unknotting of the catheter
Chopra et al. (2004) [11]	25 Y/F	Bithalamic glioma and hydrocephalus	2 months	Shunt malfunction	Excision of knotted catheter segment
Woerdeman and Hanlo (2006) [12]	10 days/M	Chiari malformation and myelomeningocele	3 days	Shunt malfunction	Unknotting of the catheter
Eftekhar and Hunn (2008) [13]	3.5 Y/M	Hydrocephalus	39 months	Shunt malfunction	Unknotting of the catheter
Mohammed et al. (2011) [9]	14 Y/M	Congenital hydrocephalus	NA	Shunt malfunction	Shunt revision
Borcek et al. (2012) [3]	3 Y/M	Head injury	34 months	Shunt malfunction	Shunt revision
Mohindra and Sharma (2012) [10]	10 Y/M	Congenital hydrocephalus and Crouzon's syndrome	7 Years	Incidental	Nil
Present case	8 Y/M	Congenital calvarial defect and hydrocephalus	2 months	Shunt infection and malfunction	Shunt revision

NA: no available data.
